# 4-weekly avelumab plus axitinib in patients with metastatic renal cell carcinoma

**DOI:** 10.1038/s44276-026-00224-y

**Published:** 2026-05-08

**Authors:** Naveen S. Vasudev, Umair Aleem, Kathryn Humphries, Manon Pillai, Ben Pickwell-Smith, Christy Ralph, Satinder Jagdev, Joanne Simpson, Helen Dearden, Tom Waddell

**Affiliations:** 1 Department of Medical Oncology, St James’s University Hospital, Leeds, UK; 2 Department of Medical Oncology, Christie Hospital, Manchester, UK

## Abstract

**Background:**

800 mg avelumab, given every 2 weeks, in combination with axitinib, is an approved first-line treatment option for patients with metastatic renal cell carcinoma (mRCC). Less frequent administration of avelumab would be preferable to patients and service providers but has not been previously explored.

**Methods:**

We retrospectively analysed the clinical outcomes of patients from two academic centres who received 4-weekly avelumab plus axitinib between Oct 2019 and Oct 2023. Patients in cohort 1 (C1) were treated with 4-weekly avelumab from the start of therapy. Patients in cohort 2 (C2) initially received standard 2-weekly avelumab and were later switched to 4-weekly infusions. Overall response rate (ORR), progression-free survival (PFS), overall survival (OS) and toxicities leading to dose reductions or cessation were evaluated.

**Results:**

We identified 94 patients in total, 46 patients in C1 and 48 patients in C2. The majority (56%) of patients in both cohorts had IMDC favourable-risk disease. The ORR in C1 was 53% and the median PFS was 22.1 months. In C2, the median time to switch was 4.9 months (range 1.8–35.6) and median time on 4-weekly avelumab was 16.7 months (range 0.9–39.1). Toxicity was consistent with prior reports. Limitations include retrospective design.

**Conclusions:**

These preliminary data support prospective evaluation of modified avelumab scheduling in patients with mRCC. Reducing treatment burden stands to improve patient quality of life and lower associated costs.

## Introduction

Standard frontline treatment for patients with metastatic renal cell carcinoma (mRCC) involves the use of checkpoint inhibitors (CPI), given in combination with each other, or in combination with a vascular endothelial growth factor receptor targeted tyrosine kinase inhibitor (VEGFr TKI) [1].

The VEGFr TKI, axitinib, in combination with the PD-L1 targeted CPI, avelumab, represents one such example. In the Phase III JAVELIN Renal 101 study, axitinib plus avelumab was associated with a significant improvement in progression-free survival (PFS) and overall response rate (ORR) in comparison to sunitinib [2]. Overall survival favoured avelumab plus axitinib but did not, however, reach statistical significance [3]. The combination is approved by both the FDA and EMA. In England and Wales, it is approved for treatment-naïve mRCC patients across International Metastatic Database Consortium (IMDC) risk groups and represents the only CPI-TKI combination available to patients with favourable-risk disease.

Avelumab 800 mg is standardly given intravenously every 2 weeks, alongside 5 mg bd of axitinib. The optimal dose and scheduling of CPI in cancer medicine remains poorly understood and, increasingly, concerns are being raised that patients may be being overtreated with these agents [4–6]. Given the financial cost of CPI, their associated toxicity, and the impact of frequent attendance for treatment on patients’ quality of life, it is critical that opportunities to reduce treatment burden are explored. A number of clinical trials have looked at or are underway examining reduced dose, extended interval and/or early cessation of CPI [7–10] although, to our knowledge, none involving avelumab.

Prompted by the COVID-19 pandemic, amongst patients with mRCC starting axitinib plus avelumab, we began to employ an extended interval dosing strategy, whereby patients received avelumab 800 mg every 4-weeks, either up-front or after an initial period of standard 2-weekly dosing. Here, we report our experience and patient outcomes.

## Patients and methods

Patients with untreated mRCC, with measurable disease, any histological type, any IMDC risk group, starting axitinib plus avelumab and who, at some point in treatment, received 4-weekly avelumab infusions, were eligible for the study. All patients were treated in one of two large academic centres in the UK, which provide regional renal cancer services across large geographic areas. Data were collected retrospectively from electronic patient health records.

Patients in cohort 1 received 4-weekly 800 mg avelumab with axitinib 5 mg bd from the start of treatment. Patients in cohort 2 started standard 2-weekly 800 mg avelumab and were later switched to 4-weekly dosing if the following criteria were met: i) at least one on-treatment CT scan demonstrating evidence of clinical benefit (complete response (CR), partial response (PR) or stable disease (SD)), ii) the patient was clinically stable and iii) the patient was willing to switch.

Patients in both centres received in-person or remote reviews, alongside safety bloods, every 4 weeks. Contrast-enhanced CT scans were performed approximately every 12–16 weeks to assess response whilst patients were on treatment.

Tumour response was derived from radiology reports by the treating clinician and categorised as either CR, PR, SD or progressive disease (PD). Endpoints included overall response rate (ORR), progression-free survival (PFS) and overall survival (OS). ORR was defined as the proportion of patients experiencing a CR or PR following treatment. PFS was calculated from the time of treatment initiation to disease progression or death from any cause; OS was calculated from the time of treatment initiation to death from any cause or censored at the time of last follow up. The estimated median PFS and OS were calculated using the Kaplan–Meier method. Statistical analyses were conducted using IBM SPSS Statistics (version 30.0).

## Results

We identified 94 patients, initiating treatment between October 2019 and October 2023. Baseline patient characteristics are presented in Table 1. Median follow-up for the entire cohort was 30.4 months. Most patients had clear cell RCC (91%) and had undergone prior nephrectomy (78%). According to IMDC criteria, the majority (56%) had favourable risk disease. Patient characteristics were well matched between C1 and C2.

**Table 1:** Patient characteristics.

	ALL	Cohort 1	Cohort 2
	*n* = 94	*n* = 46	*n* = 48
Sex	68 M:26 F	31 M:15 F	37 M:11 F
Age median (range)	67 (45–83)	65 (45–83)	69 (45–80)
ECOG performance status			
0	65 (69)	26 (57)	39 (81)
1	27 (29)	18 (39)	9 (19)
2	2 (2)	2 (4)	0 (0)
Histology			
Clear cell	86 (91)	42 (91)	44 (92)
Papillary	3 (3)	3 (6)	0 (0)
Chromophobe	2 (3)	1 (3)	1 (2))
Unclassified	3 (3)	0 (0)	3 (6)
Prior nephrectomy (%)	73 (78)	36 (78)	37 (77)
IMDC risk group			
Favourable	53 (56)	26 (57)	27 (56)
Intermediate	31 (33)	13 (28)	18 (37)
Poor	10 (11)	7 (15)	3 (7)
Sites of metastases			
Lung	52 (55)	25 (54)	27 (56)
Liver	8 (9)	4 (9)	4 (8)
Bone	25 (27)	13 (28)	12 (25)

### Cohort 1

Patients in C1 (*n* = 46) received 4-weekly avelumab from the start of treatment. Median follow-up was 27.4 months. At the time of data cut-off, 23 (50%) had stopped treatment and 15 (33%) had died. Reasons for treatment discontinuation were disease progression (35%) and treatment-related toxicity (13%). One patient stopped treatment due to being rendered in a radiological CR following deferred nephrectomy, after 34 months on therapy.

**Fig. 1:**
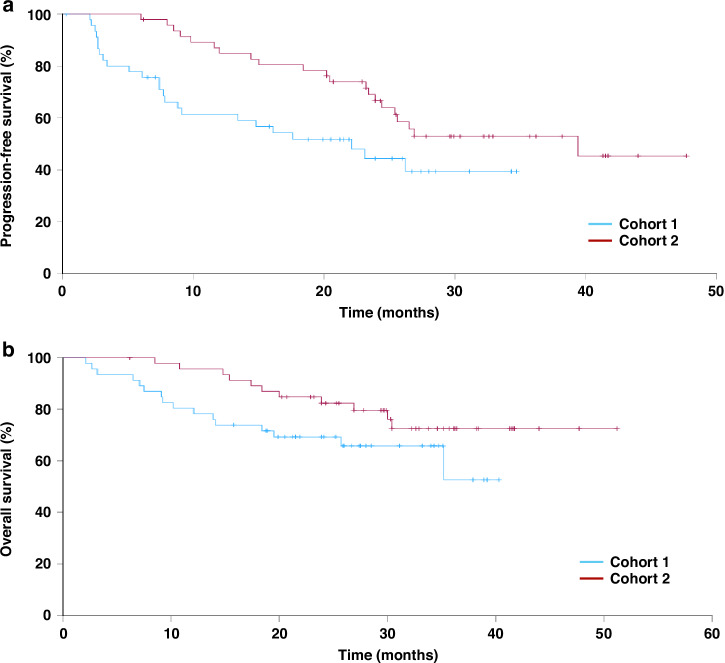
Kaplan-Meier estimates of Progression-free and Overall survival by patient cohort. Progression-Free survival (**a**) and Overall survival (**b**). Patients in cohort 1 received 4-weekly avelumab from start of treatment. In cohort 2, patients switched from standard scheduling to 4-weekly dosing following at least one on-treatment CT scan demonstrating benefit (CR/PR/SD).

Amongst the 42 evaluable patients, ORR was 53% (Table 2). The proportion of patients with progressive disease as best response was 15%. Amongst the subset of patients with IMDC favourable-risk disease, the ORR was 68% (Table 2). Median PFS was 22.1 months (95% CI 11.6–32.6). The PFS rate at 12-months was 61% (Fig. 1a). Overall survival is shown in Fig. 1b. Median OS was not reached. Landmark 1-year OS rate was 78% and 2-year OS rate was 69%.

**Table 2:** Overall response rates in Cohort 1.

	All *n* = 42^a^	Favourable *n* = 25	Int/Poor *n* = 17
Overall response rate	**22 (53)**	**17 (68)**	**5 (30)**
Best overall response *n* (%)			
Complete response	2 (5)	2 (8)	0 (0)
Partial response	20 (48)	15 (60)	5 (30)
Stable disease	13 (31)	7 (28)	6 (35)
Progressive disease	7 (16)	1 (4)	6 (35)

^a^4 patients not evaluable (no scans performed after baseline because of early toxicity or clinical decline necessitating cessation of treatment).

All patients started axitinib at 5 mg bd, except for 3 patients who started with an elective dose reduction (3 mg bd). At least one dose reduction of axitinib was required in 10 (22%) patients and at least one escalation of dose was performed in 3 (7%) patients. Reasons for axitinib dose reduction were hypertension (*n* = 4), diarrhoea (*n* = 2), oral mucositis (*n* = 2), fatigue (*n* = 2), reduced appetite (*n* = 1) and nausea (*n* = 1). CPI-related adverse events of any grade were reported in 11 (24%) patients and two (4%) required high dose steroid (≥40 mg prednisolone) (one grade 3 hepatitis and one grade 3 pancreatitis).

Amongst patients receiving subsequent therapy (13/23; 57%), the most commonly used regimens were cabozantinib (*n* = 5), lenvatinib plus everolimus (*n* = 4) and tivozanib (*n* = 3).

### Cohort 2

Patients in C2 initially received standard 2-weekly avelumab (plus axitinib) and later switched to 4-weekly dosing. Before switching, all patients were required to have had at least one on-treatment CT scan demonstrating evidence of benefit (CR (8%), PR (77%) or SD (15%)). The timing of the switch varied, with a median time to switch of 4.9 months (range 1.8–35.6). Most patients (*n* = 31; 66%) were switched within the first 12 m. Median follow-up from start of treatment was 32.9 months.

At the time of data cut-off, 23 (48%) patients had stopped both drugs and 2 (4%) patients had stopped avelumab only. Reasons for stopping both drugs were disease progression or death (91%) and toxicity (4%). One patient achieving a CR stopped treatment electively after 2 years on treatment.

**Fig. 2:**
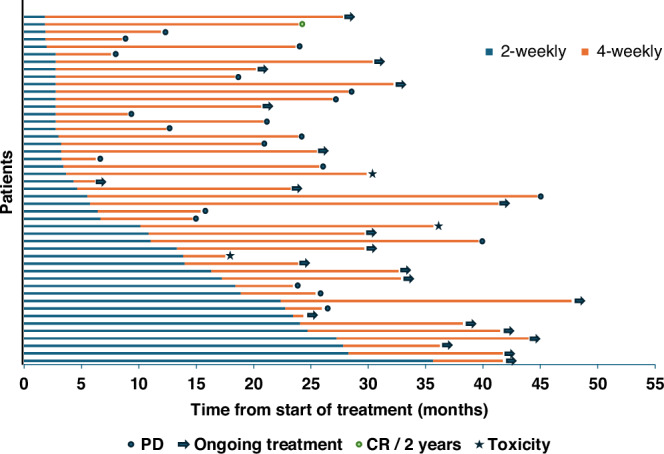
Timeline of patients in cohort 2, illustrating initiation of standard avelumab initially in all patients (blue line), before switching to 4-weekly scheduling (red line).

The median time on 4-weekly avelumab was 16.7 months (range 0.9–39.1) (Fig. 2). Median PFS was not reached, with 12 m PFS rate of 85% (Fig. 1a). Median OS was also not reached. Landmark 1-year OS rate from the start of treatment was 94% and 2-year OS rate was 82% (Fig. 1b).

Fifteen patients sequenced to second-line therapy, which included lenvatinib plus everolimus (*n* = 6), cabozantinib (*n* = 4) and single-agent everolimus (*n* = 1).

## Discussion

To our knowledge, this is the first report to describe modified scheduling of avelumab, in combination with axitinib, in patients with mRCC. Our findings provide supportive evidence for less frequent dosing of this CPI, reinforcing broader efforts to optimise the delivery of this class of drug in the clinic.

The 53% ORR, median PFS of 22.1 months and 1- and 2-year OS rates of 78% and 69% respectively amongst patients in C1 of our study are comparable with prior reports employing standard 2-weekly avelumab dosing. The phase III Javelin Renal 101 trial reported an ORR of 59.7%, median PFS of 13.9 months and median OS of 44.8 months [2, 3]. In a UK-based real-world retrospective analysis of 130 patients with mRCC, the ORR was 62%, median PFS was 13.5 months and the OS rate at 12 and 24 months was 81.5% and 65.3%, respectively [11]. In a retrospective real-world Japanese study (J-DART) (*n* = 48), the ORR was 48.8%, median PFS 15.3 months, and median time to treatment discontinuation was 15.2 months [12]. Amongst 104 patients treated in the AVION study, the ORR was 46% and the 12-month OS rate was 82.7% [13]. Finally, in the real-world ambispective RAVE-Renal study of 125 patients, the ORR was 44.3%, the median PFS was 15.0 months and the 1-year OS rate was 71.2% [14]. It should be noted that the proportion of patients with favourable-risk disease varies considerably between these studies (16.7–39.0%) and represented the majority (56%) of patients in the current cohort.

Patients in cohort 2 switched to 4-weekly avelumab following initial demonstration of clinical benefit using standard scheduling and presents an alternative way by which to explore modified scheduling (akin to a switch-maintenance strategy). Since all patients achieved at least SD as best response in this group, outcomes were superior to those in cohort 1, as expected. Outcomes were further biased given that some patients were switched after more than 18 months on standard scheduling, pre-selecting a group with established durable disease control. Despite these limitations, we believe the excellent outcomes observed amongst this cohort provide reassurance that a switch in schedule is not associated with a demonstrable loss of efficacy.

Modified scheduling of anti-PD-L1 CPIs would not be expected to significantly modify their toxicity profile and, although robust adverse event (AE) recording was limited by the retrospective nature of our study, we did not observe any clear differences when compared to a standard schedule. For example, treatment-related discontinuation rates of 7.6% in JAVELIN-101 and 10.4% in the J-DART study are comparable to the 13% reported here [2, 12]. Similarly, the 4% rate of high-dose steroid use is similar to the 4–11.1% reported elsewhere [2, 12, 14].

In comparison to avelumab, other CPI have been approved to be given using less frequent dosing intervals. Atezolizumab, another PD-L1 targeted agent, can be given Q4W, Q3W or Q2W. Nivolumab and pembrolizumab, both targeting PD-1, can be given Q4W/Q2W or Q6W/Q3W, respectively. One reason for this difference may be related to avelumab’s relatively short half-life, at approximately four days, following a 10 mg/kg dose [15], as compared to nivolumab (~25 days)[16], pembrolizumab (14–22 days)[17], and atezolizumab [18] (~27 days). Pharmacodynamic efficacy of CPI may, however, be better informed by measures of T cell receptor occupancy (RO). Studies of RO rates using nivolumab, for example, indicate sustained target occupancy >70% for at least 8 weeks, when serum levels are undetectable, and that saturation can be achieved using significantly lower doses than those routinely used in the clinic [16, 19]. Similarly, RO rates following avelumab at either 3 mg/kg or 10 mg/kg were 90% or greater at the end of its 2-week dosing interval, and may similarly be sustained for much longer, despite being cleared from the circulation [15]. Such data provide the rationale for on-going CPI optimisation studies, exploring extended interval dosing or reduced dose [8].

Reducing overtreatment with CPI would bring numerous benefits. For patients, extended interval dosing would be expected to lead to improved quality of life, by reducing the number of hospital visits and requirement for pre-treatment assessment. The financial toxicity of CPIs is also significant, with, for example, the list price of one year of avelumab in England to be approximately £80,000 [20]. Thus, the potential savings to health systems, with finite and often scarce resources, are significant [21], not just in drug costs but also the associated burden on pharmacies and treatment delivery units. The recent introduction of subcutaneous formulations of CPIs such as atezolizumab, nivolumab and pembrolizumab, represents a major step forward in enhancing the convenience and efficiency with which these drugs are delivered, and further widens the gap between those requiring more frequent, intravenous, administration.

The retrospective nature of the dataset and the relatively small number of patients represent limitations of our study. Retrospective response assessment in real-world studies such as ours is reliant on clinician judgement and responses were not RECIST-defined nor formally confirmed. Recording of AEs represents another limitation of real-world datasets, which we acknowledge. Although the toxicity of the combination, using either 2-weekly or 4-weekly avelumab, was dominated by axitinib-related issues, ideally a prospective comparison of safety and tolerability should be undertaken. PD-L1 tissue expression was not routinely undertaken and, hence, association of outcomes with PD-L1 status could not be determined. The proportion of patients in our cohort with favourable-risk disease (56%) was more than double that in the pivotal phase III trial (22%) [2] and is also higher than that observed in previous reports examining standard 2-weekly scheduling, which may have affected outcomes.

## Conclusions

In conclusion, we provide the first data reporting the activity of 4-weekly avelumab, in combination with axitinib, amongst patients with mRCC. Our findings support formal prospective comparison of standard versus extended interval avelumab dosing.

## Data Availability

Data is available on request to the corresponding author.
